# The Effect of Sintering Temperature on the Densification and Magnetic Performance of NiCuZn-Ferrites (CuO: 0–6 wt.%)

**DOI:** 10.3390/ma17102293

**Published:** 2024-05-13

**Authors:** Stefanos Zaspalis, Georgios Kogias, Vassilios Zaspalis

**Affiliations:** 1 Chemical Process and Energy Resources Institute, Center for Research and Technology-Hellas, 57001 Thessaloniki, Greece; kogias@certh.gr (G.K.); zaspalis@certh.gr (V.Z.); 2 Department of Chemical Engineering, Aristotle University of Thessaloniki, University Campus, 54124 Thessaloniki, Greece

**Keywords:** NiZn ferrites, copper oxide, magnetic properties, losses

## Abstract

This article reported on the effect of Cu-content and sintering temperature on the magnetic permeability and power losses of monolithic iron-deficient NiCuZn-ferrite components with low Cu-contents aimed to be used for power applications at frequencies up to 1 MHz. In particular $\left[{\left({Ni}_{\alpha }{Zn}_{b}\right)}_{1-x}{Cu}_{x}\right]{Fe}_{1.9}O_{4}$ ferrite compositions are investigated with a constant Ni/Zn atomic ratio a/b = 0.9 and 0 < x < 0.017. As found, the addition of Cu enables the achievement of good magnetic performance at lower sintering temperatures and, therefore, lower production cost. At all Cu-contents, the initial permeability as a function of the sintering temperature passes through a maximum above which structural deterioration due to asymmetric grain growth occurs. The temperature at which this maximum permeability occurs depends on the Cu content and coincides with the achievement of the maximum density of 5.1–5.2 g cm^−3^ (relative density ~97%). At Cu-contents x = 0.006–0.012 and sintering temperatures 1200–1100 °C power losses (tan(δ)/μ at 1 MHz, 25 °C) οf 50 × 10^−6^ could be achieved and initial permeabilities (10 kHz, 0.1 mT, 25 °C) of around 400 with very good frequency and temperature stability. At CuO content higher than 4 wt.% (i.e., x > 0.012) and sintering temperatures higher than 1150 °C, pronounced microstructural disturbances due to asymmetric grain growth result in low permeabilities and high losses. It is suggested that at low CuO contents and low sintering temperatures, the densification enhancement may not proceed through Cu-rich phase segregation but through the creation of oxygen vacancies.

## 1. Introduction

Substituted NiZn-ferrites have been established as a well-accepted soft ferrite material family for the manufacturing of high-frequency, miniaturized, and surface-mounted electronic components [1,2]. Among those, NiCuZn-ferrites form an important category. The main driver for the introduction of Cu in NiZn-ferrites was the necessity for the development of low-sintering temperature materials compatible with the low melting temperatures of the electrode (usually Ag) metals employed in multilayer components [3,4]. However, Cu is also a very useful additive for the manufacturers of monolithic NiZn-ferrite components. As a raw material, Cu is cheaper than Ni. In addition, the low required sintering temperatures favor lower energy costs. Many traditional NiZn-ferrite manufacturers of monolithic components for high-frequency power applications are, therefore, introducing Cu in the materials in order to reduce production costs. Low sintering temperatures with the simultaneous achievement of high densities and limited grain growth can also be achieved by relatively recently investigated techniques such as flash and spark plasma sintering [5,6]. However, the large-scale application of these techniques for the mass production of ferrites has to be developed and techno-economically addressed against the currently used conventional sintering. 

The effect of copper on the morphology and the magnetic properties of NiZn-ferrites has been reported in the literature [7]. Having the development of materials able to be sintered at around 900 °C as the primary goal in the great majority of the investigated cases, the Cu atomic content was much more than 10% of the total divalent cations (Ni, Zn, Cu) [8,9,10]. Such high Cu contents cannot be afforded in the development of monolithic power ferrites where power loss density is an important performance indicator and needs to be kept as low as possible. The presence of Cu-rich phases often detected along the grain boundaries [9,10,11] indicates that, at high Cu contents, solubility limits may be exceeded and that the densification and grain growth processes during sintering may be significantly different when compared to those at low Cu contents. 

The effect of Cu on the intrinsic magnetic properties of NiZn-ferrites has been studied and explained on the basis of the Cu^2+^–Fe^3+^ cation redistribution among the A and B sites, elucidating the significant differences depending on whether Cu substitutes for Ni at constant Zn content or at constant Ni/Zn ratio [10,12]. As in almost all polycrystalline magnetic ceramics, the magnetic performance, besides the unit cell composition and cation distribution, also depends strongly on the morphological characteristics of the microstructure. Cu is an additive that strongly affects the densification process and the microstructure development during sintering. Apparently, all effects and their degree of interaction are manifested in the final magnetic performance. To the authors’ knowledge, there are no systematic investigations showing the interaction between Cu content and sintering temperature at low Cu contents where no segregation of secondary phases appears. These are of particular interest for the development of monolithic power components for frequencies around 1 MHz. In numerous published investigations, the effects of important operational parameters such as iron content or sintering temperature and microstructure development are studied on one selected Cu content [11,13,14,15,16], while in other cases, the effect of Cu has been studied using one set of defined processing parameters [11,12,17,18]. 

In this article, iron-deficient low Cu content substituted NiZn ferrites with constant Ni/Zn ratio and iron deficiency ($\left[{\left({Ni}_{\alpha }{Zn}_{b}\right)}_{1-x}{Cu}_{x}\right]{Fe}_{1.9}O_{4}$, a/b = ct. = 0.91–0.92, 0 < x < 0.017) are sintered at temperatures between 1000 and 1200 °C. The interaction between Cu content and sintering temperature and the influence on microstructural development and magnetic performance is investigated. 

## 2. Experimental

**Figure 1:**
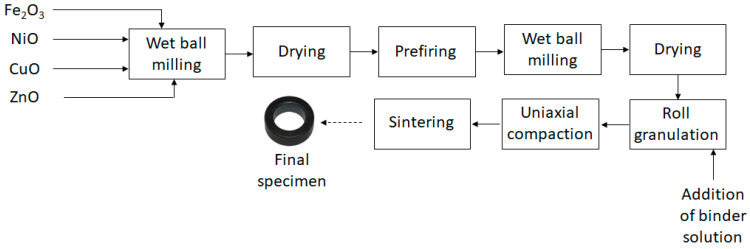
Process flow diagram of the synthesis of NiCuZn-ferrite specimens.

Polycrystalline iron-deficient NiCuZn-ferrites are synthesized using the solid-state reaction method. A process flow diagram is shown in Figure 1. The appropriate amounts of raw materials—Fe_2_O_3_, NiO, CuO, and ZnO (all Merck analytical grade)—are proportionated according to the chemical composition and weighted for the synthesis of a 500 g batch. They are subsequently wet mixed for 3 h in a laboratory ball mill with 500 mL distilled water and 3.6 kg steel balls with diameters 13–16 mm. The product is dried in air and prefired for 2 h at 890 °C in air. The prefired product is then subjected for another 3 h to a second milling step under the same conditions as those described before. No extra doping elements are added to the basic composition. The prefired and milled powder is dried and granulated in a roll granulator with the addition of 10 wt.% (on a solids basis) of an aqueous binder solution containing 2 wt.% Tylose (Tylose MH 300 P2 Shin Etsu, Venlo, the Netherlands). The granulated powder is then uniaxially compacted with compaction pressures of 100 × 10^5^ Pa to ring-shaped specimens with an outer and inner diameter of 14 and 9 mm, respectively, and a height of 5 mm. Sintering of the specimens occurs at temperatures between 1000 and 1200 °C for 3 h under air atmosphere. Six basic compositions are synthesized at constant Fe content (i.e., 1.9 atoms per chemical formula), constant Ni/Zn ratio (Ni/Zn (at.) ≈ 0.91–0.92), and Cu content that, as CuO in the raw material mixture, varied between 0 and 6 wt.% with step 1 wt.%. The corresponding name codes, raw material contents, and chemical formulas are shown in Table 1.

Particle size analysis of the powders is performed with laser scattering (Mastersizer S, Malvern, UK). Prior to analysis, all powder samples are dispersed in water and subjected to identical ultrasonic treatment. The d_10_, d_50_, and d_90_ diameters reported refer to the mean diameter below, which lies at 10%, 50%, and 90% of the volume of the sample, respectively. Specific surface areas (S_BET_) are determined by N_2_ adsorption–desorption (Autosorb 1, Quantachrome, Boynton Beach, FL, USA), while X-ray analyses are performed using CuKa radiation (Bruker D8 Advance, Leipzig, Germany). Morphological examination of the polycrystalline specimens is performed with a scanning electron microscope (SEM, JEOL JSM-IT500, Tokyo, Japan) equipped with energy dispersion analysis of X-rays (EDS), on thermally etched and gold sputtered specimens. The densities of the sintered specimens are determined by the Archimedes method, and the dilatometric experiments are performed with a Netzsch (DIL 402 PC, Selb, Germany) dilatometer on disk-shaped specimens of identical density. The magnetic properties of the specimens are measured at temperatures between 25–160 °C, using an impedance-gain analyzer (E4991A, Agilent Technologies Inc. Kobe, Japan) equipped with an oscilloscope (MDO4034C, Tektronix Inc. Beaverton, OR, USA), a power amplifier (2200L, Electronics and Innovation, NY, USA) and a frequency generator (33500B, Keysight, Santa Rosa, CA, USA), on specimens wound with copper wire so to form toroidal inductors.

## 3. Results and Discussion

**Figure 2:**
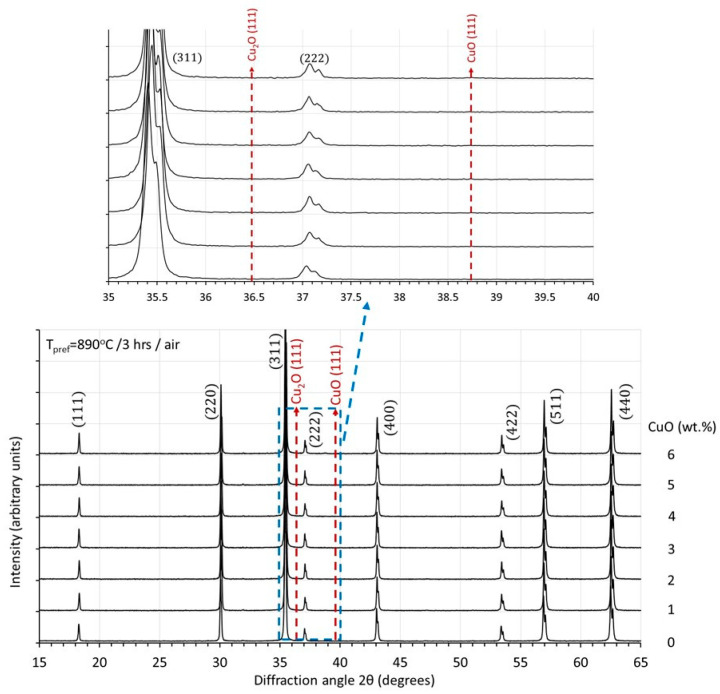
X-ray diffraction patterns of prefired samples with different Cu content.

After prefiring, all samples were investigated with X-ray diffraction. The results are shown in Figure 2. 

After prefiring at 890 °C, all samples already contain only the cubic spinel phase. No indications are present of unreacted raw materials or of the existence of secondary Cu-rich phases. In Figure 2, the diffraction angles of the most intense peaks of CuO and Cu_2_O are shown. From the X-ray diffraction results, it can be, therefore, stated that formation reactions are completed and that Cu has been integrated into the spinel lattice. 

**Figure 3:**
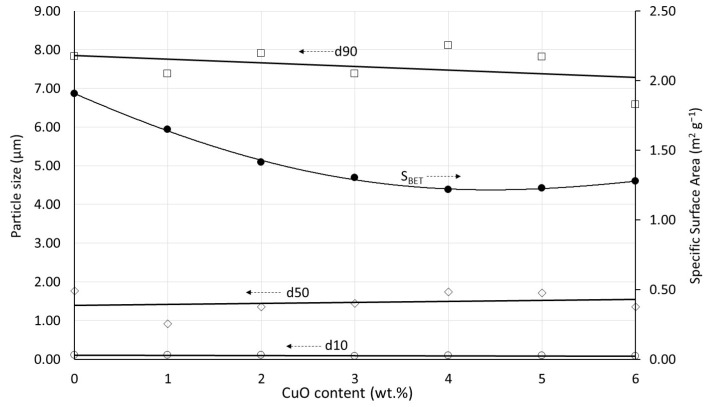
Characteristic particle sizes and specific surface areas of the prefired and milled powders as a function of the Cu content.

The prefired powders are subsequently subjected to an identical ball milling process and characterized for their average particle sizes and specific surface area. The results are shown in Figure 3.

The characteristic particle sizes are considered very comparable. There is a slight tendency towards decreased specific surface with increasing Cu content, which should be attributed to the presence of Cu. At the prefiring temperature of 890 °C, some initial sintering and neck growth have already taken place, and this is enhanced by the presence of Cu, as it will become obvious in a subsequent paragraph. 

**Figure 4:**
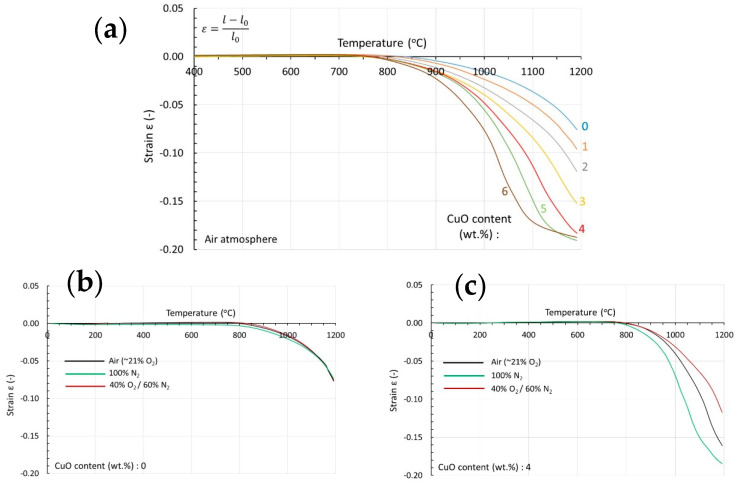
Shrinkage as a function of temperature (**a**) of specimens Cu0-Cu6 in air atmosphere, (**b**,**c**) of specimens Cu0 and Cu4 respectively, both sintered in three different atmospheres varying in the partial pressure of oxygen.

Uniaxially compacted disk-shaped specimens of the milled powders with identical densities are investigated by dilatometry. The results are shown in Figure 4a–c. 

The effect of Cu on the densification and shrinkage of NiZn-ferrites is obvious (Figure 4a). As the Cu content increases, the shrinkage rate increases. Cu acts as a densification enhancement agent. In Figure 4b, the dilatometric curves are shown for the Cu0-fired specimen in three different atmospheres varying in the partial pressure of oxygen. As can be seen, the sintering atmosphere has no effect on the shrinkage rate. Analogous results are shown for specimen Cu4 in Figure 4c. Unlike the Cu0 specimen, here, there is a detectable effect of the atmosphere. The lower the partial pressure of oxygen, the higher the shrinkage rate. Similar results are also obtained with the other Cu-containing samples. There is clearly an interaction between Cu and oxygen in the sintering atmosphere. 

**Figure 5:**
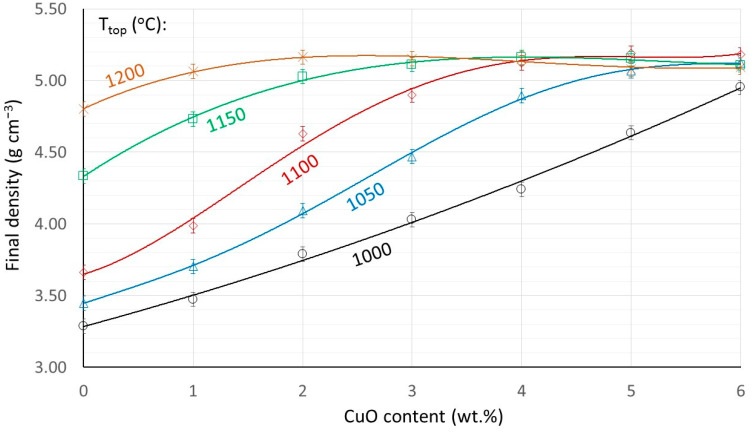
Final densities as a function of Cu content for samples sintered at different sintering temperatures. Insert: Final densities as a function of the sintering temperature for samples with different Cu contents.

Compacted ring-shaped specimens are subsequently sintered in air at temperatures from 1000 to 1200 °C. The final densities as a function of the Cu content for constant sintering temperature are shown in Figure 5. An alternative presentation as a function of the sintering temperature at constant Cu content is shown in the insert of Figure 5. 

The final density development is very systematically dependent on the sintering temperature and the Cu content. A maximum density of 5.1–5.2 g cm^−3^ seems to be the highest achievable density under all conditions. Based on the chemical formulas shown in Table 1 and the average unit cell sizes determined by X-ray diffraction, the theoretical densities are estimated to be between 5.2 and 5.25 g cm^−3^. Therefore, under the conditions of stable final densities, shown in Figure 5, and accounting for possible experimental errors of the Archimedes method, the residual porosity is estimated to be below 3%. The higher the sintering temperature, the lower the Cu content at which this density is achieved. 

**Figure 6:**
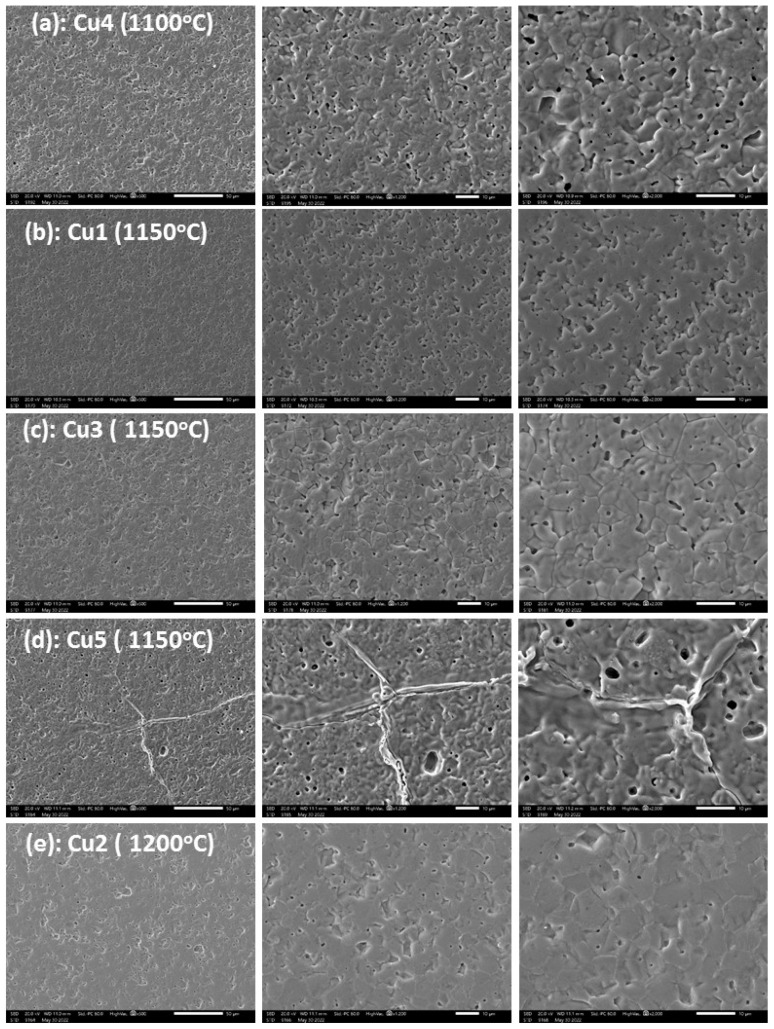
Scanning electron microscope images of selected specimens with different Cu content sintered at different temperatures.

Indicative SEM microstructure images of selected specimens are shown in Figure 6. Samples Cu1 (Figure 6b), Cu3 (Figure 6c), and Cu5 (Figure 6d) refer to the same sintering temperature (1150 °C) at different Cu contents. The progress in densification and simultaneous grain growth with increased Cu content is obvious. No exaggerated grain growth is observed as long as the density is lower or approaches ~5.2 g cm^−3^. Sample Cu5 (1150°), which reaches the maximum density already at 1100 °C, shows serious microstructure deterioration and asymmetric grain growth. Specimens of Figure 6a (Cu4-1100 °C), Figure 6c (Cu3-1150 °C), and Figure 6e (Cu2-1200 °C) have all the same final density of 5.2 g cm^−3^ and as will become obvious from a subsequent paragraph, they represent the maximum achieved permeability at each sintering temperature. The same final density of 5.2 g cm^−3^ is also achieved by the sample in Figure 6d (Cu5-1150 °C) that exhibits pronounced asymmetric grain growth. It seems that all specimens undergo normal densification and grain growth up to a maximum density of 5.2 g cm^−3^. Prolonged sintering does not result in a density increase but in microstructure deterioration due to asymmetric grain growth. The sintering temperature at which this maximum density will be reached depends on the Cu content.

**Figure 7:**
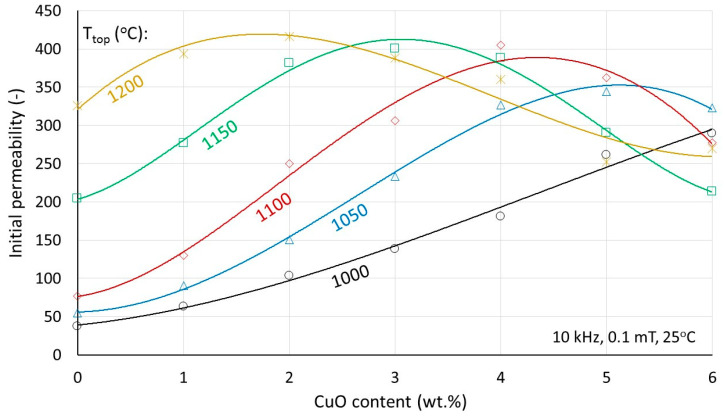
The initial permeability as a function of the Cu content for various sintering temperatures.

The initial permeabilities are shown in Figure 7. For a constant sintering temperature, the permeability increases with increasing Cu content up to a certain maximum, followed by a decrease. Comparing with the density results shown in Figure 5, it can be stated that the Cu-content corresponding to the maximum coincides very well with the Cu-content at which the maximum density of 5.2 is reached. Thus, the maximum achievable permeability depends on a combination of Cu content and sintering temperature. The permeability is dictated primarily by the densification caused by Cu. As densification proceeds, it increases, and when it stops and structural deterioration through asymmetric grain growth takes over, it decreases. As described earlier [19], this structural deterioration consists of compositional modulation, dislocations, and subgrain boundaries. 

As a criterion for the achievement of high permeability, there is not one optimum Cu content but more optimum combinations of Cu content and sintering temperature. Although with another basic composition (quite lower Ni/Zn ratio and therefore higher permeability) but comparable Cu contents, similar results are reported in [18] for one sintering temperature. 

**Figure 8:**
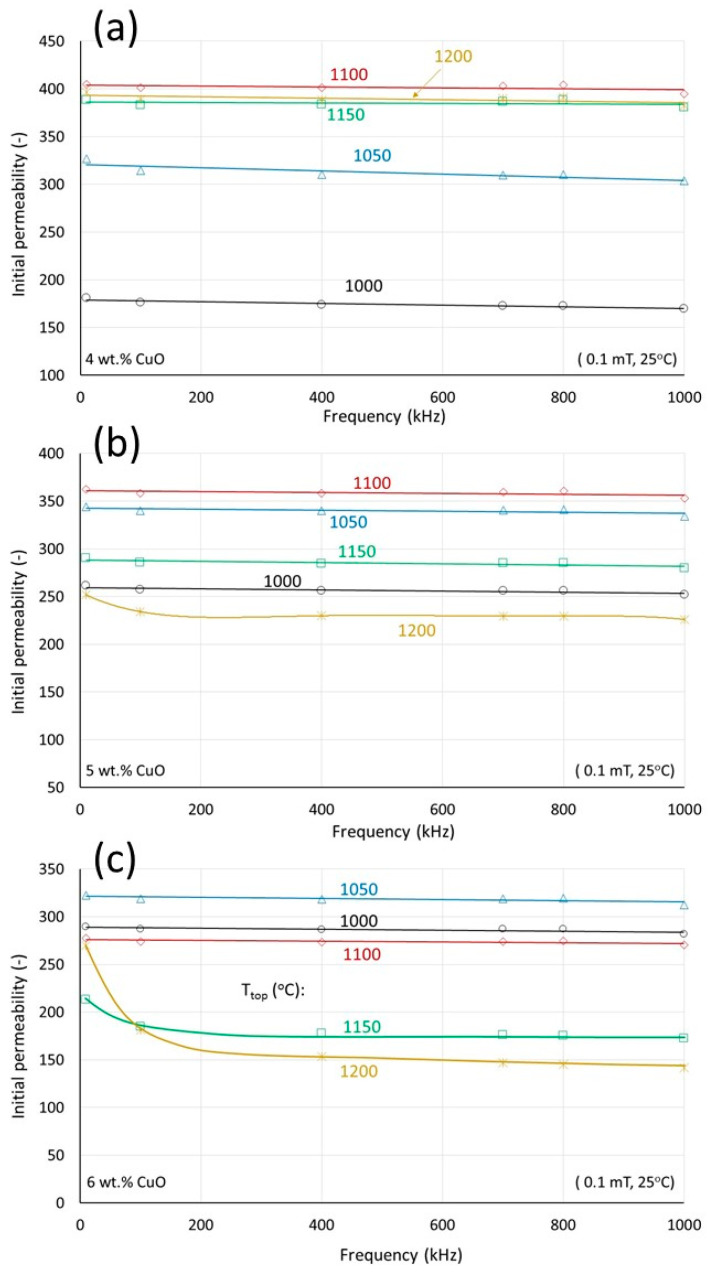
The initial permeability as a function of frequency at different sintering temperatures for samples containing (**a**) 4 wt.% CuO, (**b**) 5 wt.% CuO, and (**c**) 6 wt.% CuO.

The frequency stability of the initial permeability is very good and does not exhibit strong dependency on the densification. Independent of the absolute level, the permeability up to 1 MHz remains constant. The frequency stability of the permeability for the high CuO contents (4, 5, and 6 wt.%) is shown in Figure 8. Frequency stability is also maintained at specimens sintered after the maximum density, where the initial permeability is found to decrease with Cu content. An exception is observed only for the higher Cu-containing materials (5, 6 wt.%) sintered at the highest temperatures (1150°, 1200 °C), i.e., those at which the microstructure deterioration due to asymmetric grain growth is very pronounced. An initial decrease is observed up to 200 kHz, followed by stable behavior up to 1 MHz. Assuming that at very low fields and frequencies, the magnetization mechanism is mainly rotational. This decrease may be due to the gradual increased contribution of the domain wall movement to the magnetization. The latter is hindered by the many obstacles provided by the irregularities in the microstructures with extensive asymmetric grain growth. 

**Figure 9:**
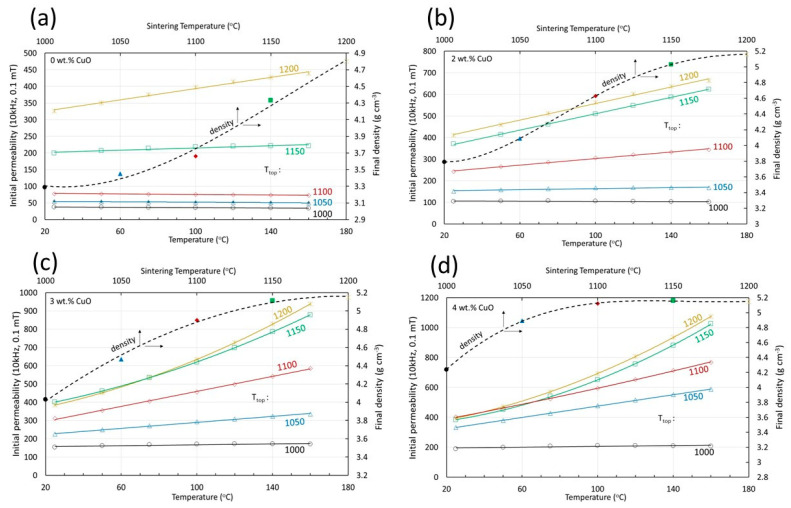
The initial permeability as a function of temperature at different sintering temperatures for samples containing (**a**) 0 wt.% CuO, (**b**) 2 wt.% CuO, (**c**) 3 wt.% CuO and (**d**) 4 wt.% CuO.

The temperature dependency of the permeability is also related to the level of densification. Selected results are shown in Figure 9. For comparison, the temperature dependency of the density is also shown for each Cu content. The higher the density, the higher the rate at which the permeability increases with temperature. As mentioned, the density is always a function of the Cu content and the sintering temperature. The same holds true for the rate of the permeability increase with temperature. In general, the permeability increases linearly with temperature. At higher Cu contents and high sintering temperatures, the increase becomes more intensive, as indicated by the parabolic curves of Figure 9c,d that correspond to sintering temperatures of 1150 and 1200 °C. 

**Figure 10:**
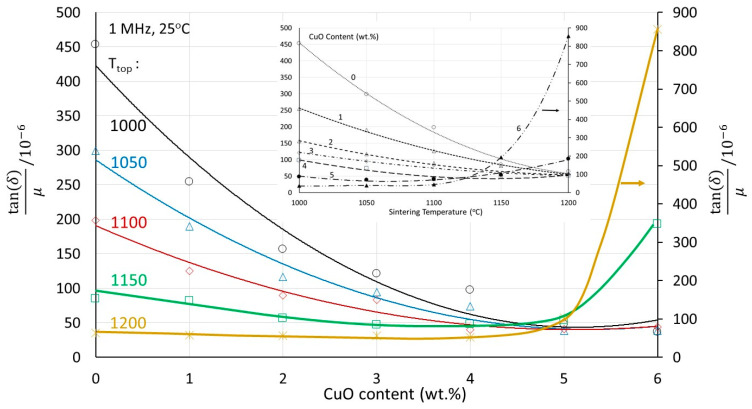
The losses at 1 MHz as a function of the Cu content for various sintering temperatures Insert: the 1 MHz losses as a function of sintering temperature for various Cu contents.

The power losses expressed as tan(δ)/μ at 1 MHz are shown as a function of Cu in Figure 10. An alternative presentation, a function of sintering temperature, is shown in the insert of Figure 10. The advantage of the introduction of Cu in lowering the power losses is obvious. At a constant temperature, the higher the Cu content, the lower the losses; at constant Cu content, the higher the sintering temperature, the lower the losses. The losses start to increase at Cu contents 5 or 6 wt.% and at sintering temperatures of 1150 or 1200 °C, probably due to pronounced microstructural disturbance caused by asymmetric grain growth. However, although Cu helps reduce the sintering temperature, it also helps to reduce the power losses. The minimum power losses achieved are ~50 × 10^−6^, and these can be achieved either with 2 wt.% CuO at 1200 °C, with 3 wt.% CuO at 1150 °C, or with 4 wt.% CuO at 1100 °C. It is not a coincidence that, as shown in Figure 7, these are also the conditions where the maximum initial permeability is reached. Unlike the initial permeability, the losses are quite stable and start increasing upon pronounced microstructural deterioration. 

From the results shown in previous paragraphs, it can be concluded that for the manufacturing of monolithic NiZn-ferrite-based power components, the CuO content should stay below 5–6 wt.% and the sintering temperature below 1150 °C. In addition, as found, the main effect of Cu is not the achievement of even better magnetic properties, at least as far as initial permeability and losses are concerned, but the reduction of the final sintering temperature and the associated reduction of the production cost.

In the literature, it is suggested that the mechanism through which Cu enhances the densification of NiZn-ferrites is liquid phase sintering [7,10,13,20]. The origin and the mechanism of creation of the liquid phase are under discussion. It is suggested that CuO segregates at the grain boundaries as a secondary phase. Cu-rich phases could be detected by the electron microscope at grains, grain boundaries, and triple points [10,11,15]. All samples were very rich in Cu, and in the formula, AFe_2_O_4_ occupied more than 15 at.% of the divalent A cations. It is also mentioned that Cu segregation along the grain boundaries occurs when the Cu content, as expressed previously, exceeds about 15 at.%. The samples presented in this study have a much lower Cu content that does not exceed 1.7 at.% of the divalent cations. The segregation of CuO alone, with a melting temperature of 1326 °C, does not provide sufficient explanation for the densification enhancement that starts already at 800 °C, as shown in Figure 4a. Moreover, it cannot account for the strong dependency of the densification on the partial pressure of oxygen in the sintering atmosphere in the presence of Cu, as shown in Figure 4b,c. 

The most probable is that the reduction of CuO to Cu_2_O takes place, as suggested in [11,15,21], according to the following reaction:

(1) $$2CuO\left(s\right)\Leftrightarrow {Cu}_{2}O\left(s\right)+\frac{1}{2}O_{2}(g)$$

The previous reaction that starts at about 1030 °C [11], in combination with the existence of a eutectic point in the CuO-Cu_2_O phase diagram at 1080 °C [22], could explain both the lower temperatures and the oxygen partial pressure dependency of the densification.

**Figure 11:**
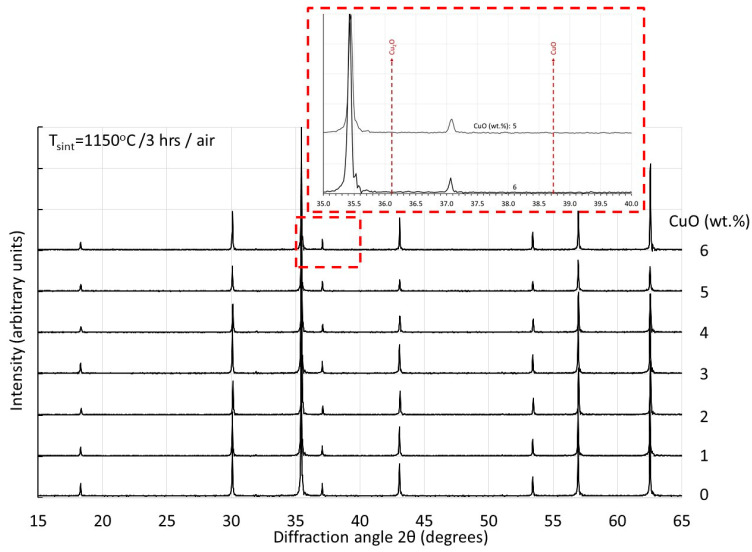
X-ray diffraction spectra of samples Cu0–Cu6 after sintering at 1150 °C.

**Figure 12:**
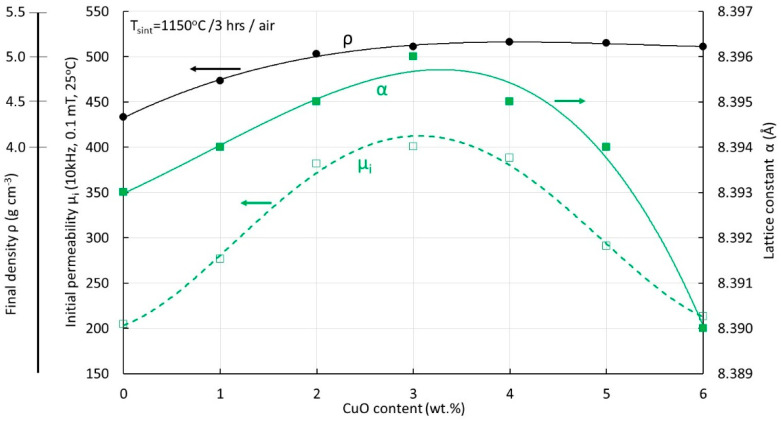
Lattice constant (α), initial permeability (μ_i_) and final density (ρ) as a function of Cu content for specimens sintered at 1150 °C.

In the sintered samples presented in this study, no indications of Cu-rich areas could be detected in EDS point analyses. For Cu1-Cu3 samples, the variations among the results of various point analyses at grains and grain boundaries were all within the statistical error of the technique used. For the high Cu-containing samples and the higher sintering temperatures, a slight tendency of increased Cu concentrations at the grain boundaries could be detected. For example, for the Cu5 sample sintered at 1150 °C, the microstructure shown in Figure 6d is characterized by pronounced asymmetric grain growth. The nominal CuO content is 5 wt.% while the average detected by overall surface analysis was 4.5 ± 0.3 wt.%. Point analysis at grain interiors indicated an average of 4.4 ± 0.3 wt.% while along the grain boundaries of asymmetrically grown grains, 4.8 ± 0.3 wt.%. X-ray analysis could not detect any Cu-rich phases in any of the sintered specimens. Similar observations have been made in [23]. Indicatively, in Figure 11, the X-ray patterns of all compositions are shown, sintered at 1150 °C. In the insert of Figure 11, a detail is shown of the pattern of the samples with the highest Cu content and pronounced asymmetric grain growth, with an indication of the diffraction angles at which the most intense peaks of Cu_2_O and CuO phases are expected. Similar results are achieved also with other sintering temperatures. The cubic lattice constants of the same sample series (i.e., after sintering at 1150 °C) are shown in Figure 12 in comparison with the values of the magnetic permeability and the final density. As the Cu content increases up to 3 CuO wt.%, the lattice constant increases. This is in agreement with the literature results [7,9,10] and indicates the substitutional integration of Cu in the spinel matrix. At higher Cu contents, the lattice constant shows a decreasing trend. The Cu content of the maximum lattice constant coincides with that of maximum permeability and achievement of the highest density. The subsequent decreasing trend in both lattice constant and permeability might be a strong indication of gradual Cu removal from the lattice towards Cu-rich regions.

It has been proven by experimental investigations that Cu ions occupy certain positions in the spinel lattice and affect intrinsic magnetic properties such as anisotropy constant and saturation flux density in a systematic way [8,12,17,18]. It cannot be excluded that at low Cu contents, far below the solubility limits and at relatively low sintering temperatures below 1150 °C, where the density and microstructure development are considered normal, CuO segregation and subsequent reduction do not take place, but densification is assisted by another mechanism. Reduction of Cu^2+^ to Cu^+^ may proceed through the creation of oxygen vacancies as charge compensating defect according to the following reaction:

(2) $$2{Cu}_{Cu}^{\times }\to 2{Cu}_{Cu}^{\prime }+V_{O}^{••}+\frac{1}{2}O_{2}(g)$$

In ionic solids with closed-packed oxygen structures, such as the spinel structure, the densification rate is determined by the diffusion rate of the large oxygen ions through anion vacancies [24,25]. This is also the reason that almost all commercially available NiZn-ferrite components for power applications are iron deficient [26]. Such a mechanism could explain the densification enhancement already from the initial sintering stage (at ca. 800 °C), where surface and grain boundary diffusion is the main mass transport mechanism, as well as the dependency of the densification rate on the partial pressure of oxygen.

Finally, it is worthwhile to mention that, as indicated in Figure 4c, sintering in Nitrogen atmospheres leads to faster densification. Although this process is not compatible with modern industrial NiCuZn-ferrite production where sintering takes place in the air, as found earlier [27], it leads to lower permeabilities and losses higher by a factor of almost 2 when compared to those achieved when sintering takes place in the air.

## 4. Conclusions

Cu enables the densification of NiZn-ferrites up to a maximum density of 5.1–5.2 g cm^−3^ at temperatures between 1050 and 1200 °C without sacrificing magnetic properties such as permeability and power losses.

For each sintering temperature, there is an optimum Cu content for the achievement of high permeability and low losses. Power losses of tan(δ)/μ of 50 × 10^−3^ with permeabilities of 400 can be achieved at various Cu contents and sintering temperatures.

For the manufacturing of monolithic NiCuZn-ferrites for high-frequency power applications, CuO contents are higher than 5 wt.% (in the raw materials) and sintering temperatures higher than 1150 °C will most probably lead to extensive asymmetric grain growth and microstructural deterioration.

It is argued that at low Cu-contents and sintering temperatures, densification is enhanced by the creation of οxygen vacancies rather than by the segregation of Cu-rich phases.

## Figures and Tables

**Table 1:** Codes of the seven NiCuZn-ferrite syntheses with corresponding contents of the raw materials and chemical formulas.

Synthesis Code	Weighted Raw Material Amounts per 100 g Material (g)				Chemical Formula (Ni_x_Cu_y_Zn_z_)Fe_1.9_O_4_ (x + y + z = 1)
	Fe_2_O_3_	NiO	ZnO	CuO	
Cu0	65.80	16.65	18.55	0	$\left({Ni}_{0.48}{Cu}_{0.0}{Zn}_{0.52}\right){Fe}_{1.9}O_{4}$
Cu1	65.80	25.19	18.01	1	$\left({Ni}_{0.47}{Cu}_{0.003}{Zn}_{0.51}\right){Fe}_{1.9}O_{4}$
Cu2	65.80	14.73	17.47	2	$\left({Ni}_{0.45}{Cu}_{0.006}{Zn}_{0.49}\right){Fe}_{1.9}O_{4}$
Cu3	65.80	14.28	16.92	3	$\left({Ni}_{0.44}{Cu}_{0.009}{Zn}_{0.48}\right){Fe}_{1.9}O_{4}$
Cu4	65.80	13.82	16.38	4	$\left({Ni}_{0.42}{Cu}_{0.012}{Zn}_{0.46}\right){Fe}_{1.9}O_{4}$
Cu5	65.80	13.36	15.84	5	$\left({Ni}_{0.41}{Cu}_{0.014}{Zn}_{0.45}\right){Fe}_{1.9}O_{4}$
Cu6	65.80	12.90	15.30	6	$\left({Ni}_{0.40}{Cu}_{0.017}{Zn}_{0.43}\right){Fe}_{1.9}O_{4}$

## Data Availability

The original contributions presented in the study are included in the article, further inquiries can be directed to the corresponding author.
